# Genomic adaptation of the ISA virus to *Salmo salar* codon usage

**DOI:** 10.1186/1743-422X-10-223

**Published:** 2013-07-05

**Authors:** Mario Tello, Francisco Vergara, Eugenio Spencer

**Affiliations:** 1 Centro de Biotecnología Acuícola, Laboratorio de Virología, Facultad de Química y Biología, Universidad de Santiago de Chile, Avenida Libertador Bernardo O’Higgins 3363, Santiago, Chile

## Abstract

**Background:**

The ISA virus (ISAV) is an Orthomyxovirus whose genome encodes for at least 10 proteins. Low protein identity and lack of genetic tools have hampered the study of the molecular mechanism behind its virulence. It has been shown that viral codon usage controls several processes such as translational efficiency, folding, tuning of protein expression, antigenicity and virulence. Despite this, the possible role that adaptation to host codon usage plays in virulence and viral evolution has not been studied in ISAV.

**Methods:**

Intergenomic adaptation between viral and host genomes was calculated using the codon adaptation index score with EMBOSS software and the Kazusa database. Classification of host genes according to GeneOnthology was performed using Blast2go. A non parametric test was applied to determine the presence of significant correlations among CAI, mortality and time.

**Results:**

Using the codon adaptation index (CAI) score, we found that the encoding genes for nucleoprotein, matrix protein M1 and antagonist of Interferon I signaling (NS1) are the ISAV genes that are more adapted to host codon usage, in agreement with their requirement for production of viral particles and inactivation of antiviral responses. Comparison to host genes showed that ISAV shares CAI values with less than 0.45% of *Salmo salar* genes. GeneOntology classification of host genes showed that ISAV genes share CAI values with genes from less than 3% of the host biological process, far from the 14% shown by Influenza A viruses and closer to the 5% shown by Influenza B and C. As well, we identified a positive correlation (p<0.05) between CAI values of a virus and the duration of the outbreak disease in given salmon farms, as well as a weak relationship between codon adaptation values of PB1 and the mortality rates of a set of ISA viruses.

**Conclusions:**

Our analysis shows that ISAV is the least adapted viral *Salmo salar* pathogen and Orthomyxovirus family member less adapted to host codon usage, avoiding the general behavior of host genes. This is probably due to its recent emergence among farmed Salmon populations.

## Background

The etiological agent of Infectious Salmon Anemia (ISA) is the Orthomyxovirus ISAV, which has had a major economic impact on Chilean and global aquaculture [1]. The genome of the ISA virus encodes for at least 10 proteins in 8 segments [2]. Most of the functions of the proteins encoded by the ISA virus have been determined by their homology with the Influenza A proteins. Segments 1, 2 and 3 encode for proteins PB1 [3], PB2 [4] and PA [5], respectively, which are homologous to the proteins that make up the replication/transcription complex in influenza A [6]. Segment 4 encodes for a protein homologous to the influenza A nucleoprotein [5,7], while segments 5 and 6 encode for proteins with membrane fusion and hemaglutinin esterase activity [8,9]. The segment number 7 encodes for two proteins homologous to matrix protein 1 (M1) and matrix protein 2 (M2) of Influenza A virus, which have Interferon I antagonist activity [10,11]. Finally, segment 8 encodes for non-structural proteins NS1 and NS2, which have shown immunosupressive activity in cell cultures [11]. Although basic information was obtained by sequence comparison, the lack of genetic tools has hampered the study of the molecular mechanism behind the virulence of ISAV.

The ISA virus was first described in 1984 [12]. Phylogenetic analyses to date have found two main groups of ISA viruses, the European and the North American [13-15], which may have diverged at the beginning of the 20^th^ century when salmon trade between Europe and America began [15].

Codon usage is a characteristic signature for each organism that reflects its evolutionary history [16]. Viral codon usage controls several viral processes such as translational efficiency and folding [17-19]. Codon usage of viral genes evolves according to their specific protein requirements [20,21]. The reduction of differences between viral and host codon usage is known as codon optimization. *In vitro*, this process increases protein expression and enhances the antigenicity of DNA vaccines [22]. Sequence analysis of viral genes from viruses with completely sequenced genome has shown that highly required viral proteins are encoded by genes optimized to the host codon usage [23]. In contrast, large-scale deoptimization, analogous to the scenario of a virus infecting a new host, had reduced protein production and virulence in the influenza A and Polio viruses [24,25]. In this work, we analyzed the host codon adaptation of the ISA virus and its relationship to the virulence and evolution of the virus. For this purpose, we used a set of European ISAV viruses with well-characterized virulence and genomic sequences. We also analyzed other viral *Salmo salar* pathogens and other Orthomyxoviruses to gain further insights into the codon adaptation of viruses to their host. Taking into account the lack of reverse genetic techniques in ISA viruses, the use of bioinformatic tools is a practical approach to understanding the biological function of the proteins encoded in the virus.

## Results

### Adaptation of ISAV to host codon usage

**Figure 1:**
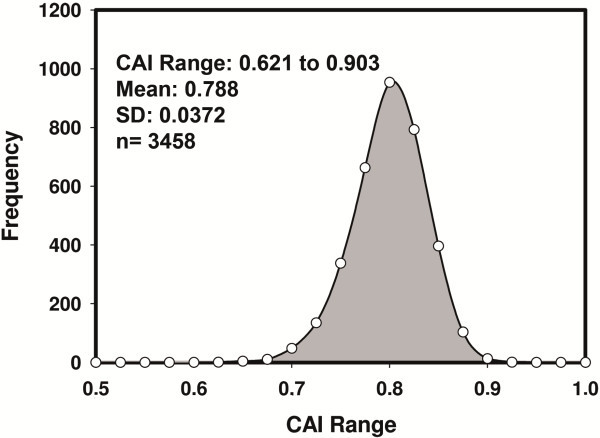
**Distribution of CAI values in the *Salmo salar *genome.** The figure shows a histogram of CAI values of the *Salmo salar* genes. The 3458 (n) analyzed have values between 0.621 and 0.903, with a mean value of 0.788 and a standard deviation (SD) of 0.0372. Approximately 70% of genes have values located within 1 SD of the mean.

The first goal of this work was to assess the adaptation of ISAV to *Salmo salar* codon usage. As an initial approach, this can be evaluated using the codon adaptation index (CAI) [26]. CAI values have bell-shaped distributions in *Salmo salar* (Figure 1). The CAI values resulting from the analysis of the 10 coding regions of the ISA virus, shared by 17 fully sequenced viral genomes in European and North American ISAVs showed that genes encoding for structural proteins (NP and M1) and antagonists of Interferon I signaling (NS1) have the highest CAI. The European ISAVs show higher values in the genes encoding the nucleoprotein, while the highest value in the North American virus is the Matrix 1 encoding gene. In both kinds of viruses, genes for proteins involved in replication (PA and PB2) and RNP traffic (NSP2) have low CAI. Among the ISAVs analyzed, Chilean isolates ISAV901 and ISAV752 have the highest CAI values in genes related to replication (PB2, and PA), structure (M1) and immunosuppression (M2), and the lowest values in genes encoding for surface glycoprotein. The North American isolate of ISAV (ISAVNA) has the highest number of genes with low CAI values (Table 1).

**Table 1:** Adaptation of ISAV genes to Salmo salar codon usage

	**CAI values of ISAV genes**									
**ISA virus**	**PB2**	**PB1**	**NP**	**PA**	**F**	**HE**	**NSP1**	**NSP2**	**M1**	**M2**
ISAV752	**0.598**	0.610	0.668	**0.591**	0.577	0.622	0.645	0.591	**0.647**	**0.612**
ISAV901	**0.598**	0.611	0.668	**0.591**	0.577	0.621	0.645	0.591	0.644	0.609
ISAV1	0.595	0.611	0.671	0.590	0.579	0.620	0.641	0.589	0.646	0.608
ISAV10	0.595	0.610	0.671	0.587	0.577	0.625	0.645	0.594	0.644	0.610
ISAV11	0.597	0.611	0.671	**0.592**	0.578	0.630	**0.646**	0.591	0.646	0.608
ISAV2	0.594	0.610	0.670	0.586	0.582	0.628	0.636	0.592	0.642	0.603
ISAV3	0.595	0.608	**0.672**	0.587	0.582	**0.635**	0.643	**0.596**	0.642	0.606
ISAV4	0.595	0.609	0.668	0.590	0.576	0.625	0.644	0.594	0.644	0.609
ISAV5	**0.598**	**0.611**	0.669	0.587	**0.584**	0.629	**0.646**	0.588	**0.649**	0.608
ISAV6	0.595	0.611	0.666	0.585	**0.584**	**0.632**	0.639	0.592	0.642	0.603
ISAV7	0.594	**0.612**	0.667	0.587	0.583	0.627	0.638	**0.595**	0.644	0.608
ISAV8	0.595	0.610	0.668	0.586	0.579	0.621	**0.647**	0.594	0.646	0.605
ISAV9	0.595	**0.612**	**0.672**	0.585	0.581	0.627	0.638	0.592	0.646	0.608
SK779	0.595	0.610	**0.672**	**0.591**	0.577	0.623	0.643	**0.596**	0.646	0.605
VIR22	0.596	0.611	0.670	0.587	0.582	0.623	0.638	0.592	0.646	0.608
VIR25	0.596	0.611	0.670	0.590	**0.583**	0.622	0.638	0.592	0.646	0.608
VIR28	0.590	0.611	0.668	0.587	0.581	**0.630**	0.640	0.592	0.642	0.603
ISAVNA	0.555	**0.612**	0.633	0.555	**0.607**	0.564	0.640	0.556	**0.656**	**0.623**

Higher and lower CAI values per segment are indicated in bold and underlined characters respectively.

To increase our understanding of the biological significance of the CAI from ISAV genes in the context of the host, these values were compared and normalized according to the scores achieved by the *Salmo salar* genes. Genes from European isolates have Z-CAI values between −6 and −3, in contrast to the ISAVNA, which has values between −7 and −3. Conversely, less than 0.45% of *Salmon salar* genes have Z-CAI values lower than −3. To determine if this behavior is shared by other viral pathogens of Salmon or other Orthomyxoviruses, we calculated the Z-CAI for the genes of the most common *Salmo salar* viruses: *Atlantic salmon* swim bladder sarcoma (ASSBS), infectious hematopoietic necrosis virus (IHNV), viral hemorrhagic septicemia virus (VHVS), infectious pancreatic necrosis virus (IPNV) and the salmon pancreas disease virus (SPDV). We also analyzed common human Orthomyxoviruses (Influenza A, B, C and Thogoto) to understand how viruses phylogenetically related to ISAV evolved in different virus-host systems. As Z-values are normalized with respect to the host genes, this score allows comparing viruses that infect different organisms.

**Figure 2:**
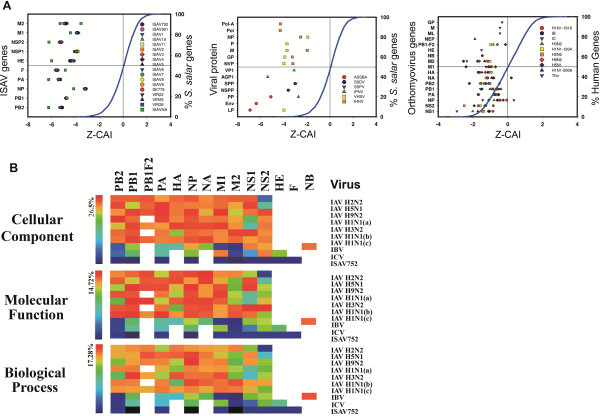
**Relation with host genes.** The figure shows the Z-CAI values for ISAV genes, the viral pathogens of *Salmo salar*, the influenza A, B, C and the Thogoto virus **(Panel A)**. The blue lines at the left, center and right figures of **panel A** show the accumulated frequency (expressed in percentage, in the right axis) of the Z-CAI values of host genes. **Panel B** shows the mimicry of the cellular function by viral genes. The Figure shows the percentage of cellular functions that contain genes with similar CAI. The classification by Blast2Go for *H. sapiens* shows a total of the 7010 biological processes, 3125 molecular functions and 1030 cellular components. For *Salmo salar* a total of 3304 biological processes, 1323 molecular functions and 643 cellular components were identified. **(a)** H1N1 2009, **(b)** H1N1 1918 and **(c)** H1N1 1934.

The results show that with the exception of ASSBS viral genes, the Z-CAI values of the genes of other viral *Salmo salar* pathogens have values closer to the mean, ranging between −7 and −5 for ASSBS, -5 and −1 for IHNV, -4 and −1 for VHVS, and between −4 and −2 for IPNV and SPDV. The IHNV and VHVS share Z-CAI values with 3.38% of the *Salmo salar* genes, while IPNV and SPDV share values with less than 0.87% of cellular genes. The human Orthomyxoviruses have Z-CAI values ranging between −3 and 0.5. The Influenza A, B, C and Thogoto virus share Z-CAI values with ~47%, ~56%, ~11%, and ~ 35% of human genes, respectively. All viral genes from human Orthomyxoviruses have lower values than the mean (negative Z-score), with the exception of neuraminidase from the Influenza B virus and the nucleoprotein of Thogoto (Figure 2A, Additional file 1: Tables S1, S2 and S3). These results show that among Orthomyxoviruses, ISAV displays the lower values of adaptation to host codon usage, escaping from the general behaviour of host genes.

To determine if ISAV is mimicking a biological process, molecular function or cellular component, we compared the CAI values of the ISAV genes to CAI values of the genes of their respective hosts according to the parameters of gene ontology. Analysis of the ISAV virus showed that less than 1% of the biological processes, molecular functions or cellular components of *Salmo salar* contain genes with CAI values similar to those of the ISA virus. On the other hand, in ISAV752, the genes that encode for NP, PB1 and M1 were the only ones that share CAI values with some other *Salmo salar* genes classified according to GO. When the viral genes of the ISAV, Influenza A, B, C and Thogoto were compared, the genes encoding for NP, M1 and PB1 were found to share their CAI values with the greatest number of genes belonging to some cellular function of their host (Figure 2B). Interestingly, the 1918 H1N1 and the 1934 H1N1 viruses shared their CAI values with genes related to a greater number of cellular functions (Additional file 2: Figure S1). This confirms that among Orthomyxoviruses, ISAV does not follow the general behavior of host genes and genes involved in cellular metabolism.

### Evolution of CAI values in ISA viruses

**Figure 3:**
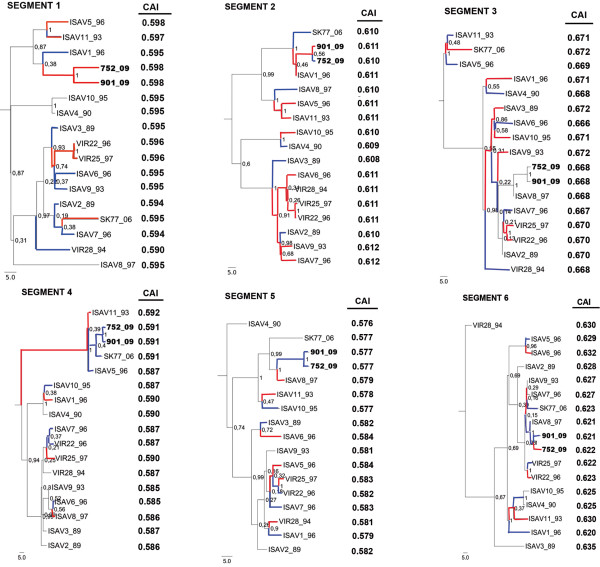
**Comparison of CAI values from segments of closely related ISAV.** The alignment was taken from a previous work of our group [46]. Red lines show the divergence that increases the CAI value and the blue lines show the divergence that decreases CAI values.

In order to determine if the evolution of each segment of ISA viruses has increased or decreased their adaptation to host codon usage, we related the CAI values of each segment to its phylogenetic relationship, focusing on the Chilean isolates. The Chilean ISAV isolates 752 and 901 increased or maintained their CAI values in segments 1, 2, 3, 4 and 5, while in segment 6, which encodes for glycoprotein HE, CAI values decreased. The closest homologue to the Chilean isolates in segments 1 and 2 is the highly virulent ISAV1 strain, while in segment 3 and 5 the closest homologue belongs to the low virulence strain ISAV8, and in segment 4, to the avirulent strain SK779 (HPR0). Segments 5 and 6, on the other hand, are closely related to the strain ISAV8 (Figure 3). If mutations in ISAV affect the translation rate, it can be expected that CAI rates among different segments will show similar variations when their genetic products interact. To determine if this relation exists, the CAI values of the ISAV were correlated.

Regarding the prediction of translation efficiency using CAI, when we use rank sum spearman’s test with a p value of 0.05 as cut off for statistical significance ten correlations were found, PB2 correlated positively with PA (ρ=0.599) and NS1 (ρ=0.485). The gene that encodes for PB1 correlated positively with F (ρ=0.536) and NS2 (ρ=0.491). The PA encoding gene correlated negatively with genes for the F protein (ρ=−0.640). Genes encoding for F and HE correlated negatively with NS1 (ρ=−0.490) and M2 (ρ=−0.570), respectively. The CAI for the M1 encoding gene correlated with M2 (ρ=0.527), while NP did not correlate with any other gene (Additional file 2: Figure S2). However when we apply the Bonferroni correction for multiple correlations and we increase the stringency reducing p-value cut off to 0.005 non statistical correlation were found. These results suggest that the evolution of most of the genes from Chilean isolates have occurred without a host codon adaptation cost, and genes encoding proteins that are predicted to interact among themselves, such as those involved in the transcription/replication complex (PA, PB2, PB1), appear to have evolved coordinating their host codon adaptation.

### Temporal evolution of codon adaptation among orthomyxoviruses and viral pathogens of salmon

**Figure 4:**
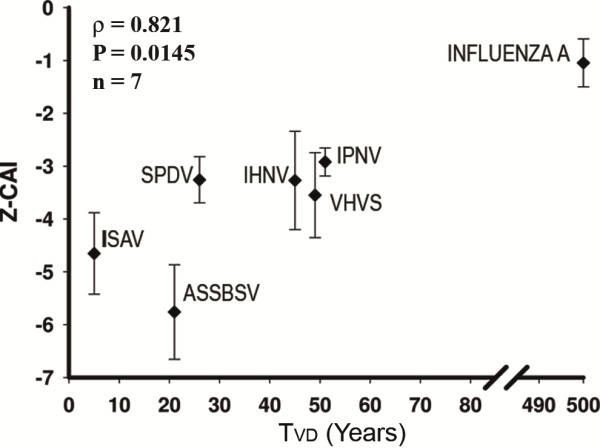
**Correlation between time of description and codon adaptation.** The figure shows the correlation between the time of description (time between the first description of the disease and the first completely sequenced genome) and the mean Z-CAI values of the genes from the ISAV, ASSBSV, IPNV, IHNV, VHVS, SPDV and influenza A viruses.

To determine if the degree of host codon adaptation of ISAV depends on the time that the virus and host have been in contact, we analyzed the correlation between CAI values of the viral genes and the duration of the virus–host relationship. However, it is impossible to determine when a virus first arose and measure the length of the relationship because viruses are in continuous evolution. As well, if we compare viruses with different numbers of genes or different hosts, the biological significance of CAI values might not be the same. To overcome these problems, we opted for a different method to determine the duration of a virus-host relationship, that of evaluating the window period. Thus, we defined the time T_vd_ as the time between the first description of the disease in Salmon farms and its molecular characterization by sequencing. The latter was chosen as an endpoint since our bioinformatic analysis cannot rule out that sequence changes occurred prior to or after viral sequencing. Thus, T_vd_ represents a short window in the history of a virus. However, if changes in codon adaptation continue over time, it can be expected that the absolute value of the codon usage adaptation index is proportional to this window period. Under this definition, the viral *Salmo salar* pathogens IPNV, SPDV, VHSV, IHNV and ASSBS, and ISAV present a singular opportunity, given that these are all RNA viruses have established dates for the first outbreaks on salmon farms: ISAV (1984) [27], IPNV(1940) [28], SPDV(1976) [29,30], VHSV(1954) [31,32], IHNV (1950) [33] and ASSBS (1975) [34]. Also Influenza A are RNA viruses which presence in human population has been estimated at least in 500 years [35]. We used the average Z-CAI values to compare different types of viruses that infect different hosts and that have different numbers of genes. The results show that the average of the Z-CAI values of the genes of the ISAV, viral pathogens of *Salmon salar* and those of Influenza A correlate significantly (ρ = 0.821, P = 0.0145, n=7) (Figure 4) with the T_vd_ values, which is maintained when the average of each of the ISA viruses is incorporated independently (ρ = 0.496, P = 0.014, n = 24), and also when the influenza A virus is eliminated (ρ = 0.426, P = 0.0419, n = 23). When only the values of the *Salmo salar* pathogens are used, a positive correlation is obtained (ρ= 0.489, P = 0.018, n= 23), which increases upon incorporating the average of the influenza A virus (ρ=0.551, P = 0.00548, n = 24).

When we evaluated changes over time in the Z-CAI values of ISAV genes, we noted that there is a correlation among ISAVs (P <0.05) between the T_vd_ and the Z-CAI values of PA (ρ = 0.475, P = 0.045, n= 18) and PB2 (ρ = 0.577, P = 0.0121, n= 18). Although last results are promissory they did not pass the statistical significance of Bonferroni correction (P <0.005). These results show that among ISAV and viral pathogens of Salmon, viruses with earlier reported dates of outbreak on Salmon farms tend to display better adaptation to host codon usage.

### Correlation between adaptation to codon use and mortality induced by ISAV infections

To determine if there is a relationship between codon use of ISAV and virulence, we analyzed the correlation between the virulence of a bounded set of the ISA viruses and the CAI values of their genes. No statistically significant correlation was observed between mortality and CAI values when comparing the CAI values of several ISA viruses with well-characterized mortality levels.

### Assessing codon adaptation of ISAVs with the arithmetic mean of codon frequency in a gene

CAI is a geometric mean among the relative adaptiveness of synonymous codons. As geometric mean is influenced by extreme values it shows a sharply bell-shaped histogram. To determine if the poor dispersion of data was due to missing correlations, we calculated the arithmetic mean of codon frequency. The arithmetic mean was normalized for the maximum and minimum values that a gene encoding for the same protein can achieve. The arithmetic mean of *Salmo salar* genes correlates well with CAI values (ρ=0.89, P=2x10^-7^, n=3458) and, as expected, shows a flatter bell-shape distribution (Additional file 2: Figure S3). In general, the arithmetic mean of codon frequency in ISAV genes shows the same behavior as CAI values (Additional file 1: Tables S4, S5, S6 and S7, and Additional file 2: Figure S4). However, ISAV genes share values of the normalized arithmetic mean of codon frequency with less than 20% of *Salmo salar* genes, and the number of *Salmo salar* genes with GO classifications increased by only 3%. Interestingly a significant negative correlation (ρ= −0.631, P=0.039, n=11) was found between the mortality rates of well-characterized ISAVs [36] and normalized arithmetic mean values from their respective PB1 genes. This correlation was not enough strong to pass the statistical significance when the Bonferroni correction for multiple correlation was applied (p = 0.005).

## Discussion

### Adaptation of ISA virus genes to *salmo salar* codon usage

Synonymous mutations are far from being silent, since they have been shown to affect a large number of functions, participating in regulating translation and RNA folding, among others [17]. Owing to the scarcity of its genetic material, a virus must develop strategies to compact its vital information in a few kilobases [37]. Through the modification of codon usage, silent mutations passively control processes such as the kinetics of translation and folding [18], and RNA packaging [38,39]. It has recently been shown that the deoptimization of codon pair usage in the influenza A and polio viruses significantly reduces the quantity of protein produced, decreasing the number of viral particles generated and thus also reducing virulence [24,25,40,41]. This strategy constitutes an effective manner of generating live vaccines against viruses [24,25,40]. In contrast, the efficacy of a DNA vaccine is directly related to protein expression, which in turn depends on codon usage [22,42]. Influenza A DNA vaccines has been made more efficient by modifying the codon usage of the genes that encode for the membrane glycoprotein HA to resemble the codon usage frequency of the host cell [21]. Codon pair optimization also increases the protein production in polioviruses, but in contrast to codon pair deoptimization, this does not affect virulence. Although the *in vitro* relationship between codon usage and virulence is well understood, it is not known whether the virus, in its natural form, modifies its codon usage with contact time with the host, or if highly virulent viruses, besides encoding for proteins with increased capacities, also possess genes highly adapted in their codon usage, so that they are effectively preprogrammed for high levels of expression. As has been described with other viruses [23], genes in ISAV encoding for highly required structural proteins (NP and M1) have higher values of adaptation to host codon usage (Table 1 and Figure 2). Our results, based on the analysis of codon usage, show that the ISAV virus is the least host-adapted Orthomyxovirus, and the second least adapted *Salmo salar* pathogen after ASSBS (Figure 2). It is not known if ISAV uses deoptimization as a biological strategy to avoid competition for cellular tRNA, similar to the strategy of the Hepatitis A virus (HAV), which uses deoptimization to increase tRNA availability and achieve adequate capsid protein folding [18,43]. Although virulence is a multifactorial phenomenon, the negative correlation between adaptation to the codon use of the PB1 gene and observed virulence is interesting. Notably, SK779 (HPR0) is an avirulent strain with similar codon use adaptation values to those in highly pathogenic viruses. Nevertheless, the pattern of codon-use adaptation values obtained with hierarchical clustering indicate that SK779 has a pattern of adaptation values similar to those of the ISAV strains 4, 8 and 10, which low levels of virulence (data not shown). Currently it is thought that the majority of ISA viruses of the Norwegian genogroup originate from the SK799/HPR0 strain [44]. Our correlations of CAI with T_d_ for the PB2 segment support this view.

### Extreme deoptimization of ISAV genes and coordinated changes

The extreme deoptimization observed could also be explained if ISAV were an emerging pathogen in *Salmo salar* populations; in effect that it is currently adapting its codon usage to the codon preference of its host. It is still a matter of debate if animal viruses have evolved to adopt to host codon preference, although is a well described phenomena in bacteriophages [45]. The results of our analysis of CAI values of ISAV isolates closely related phylogenetically (Figure 3), and the correlation of T_vd_ with the duration of the virus-host relationship (Figure 4), support this idea. Although, initially our results showed the existence of a positive co-evolution of the index values among the genes encoding the proteins that make up the viral RNA-polymerase RNA dependent complex [46] and the matrix protein (Additional file 2: Figure S2), the application of Bonferroni correction to minimize type 1 error increased the statistical significance impairing conclude the existence of a co-evolution among CAI values. Despite it, the initial results are in accordance with the need to coordinate the relative amounts of the proteins that participate in viral transcription and replication with those that constitute part of the structure of the virus. Co-evolution among interacting proteins of gene expression evaluated through CAI has been described [47]. The molecular interaction among the ISAV glycoprotein and M1 has also been described [48].

## Conclusions

To our knowledge, the results of this work show for the first time the statistical relationship among the codogenic composition of a virus and virus-host contact time (Figure 4). Our results suggest that “part of the evolution of the viral pathogens of *Salmo salar* involves increasing their adaptation to the frequency of host codon use”, and therefore also supports the notion that ISAV is an emerging pathogen, it being the least adapted viral *Salmo salar* pathogen and Orthomyxovirus to host codon usage and thus avoids the general behavior of host genes (Figures 2 and 3). Thus, the analysis of synonymous viral mutations in the context of cellular genes constitutes a complementary approach to understanding virulence mechanisms and the evolution of viral pathogens, especially those that have diverse hosts. Until reverse genetic techniques can be applied with ISAV, sequence analysis is a good strategy to assess and propose a descriptive model for the molecular mechanism behind ISAV virulence.

## Methods

The sequences of the coding regions of viral pathogens of *Salmo salar* were obtained from NCBI. Sequences of the coding regions of influenza A, B, C, and Thogoto were obtained from the NCBI viral genome database. Sequences of the coding regions of the human and *Salmo salar* genes were obtained from the Refseq database (NCBI). The list of accession numbers of the sequences used is included in supplementary information. Codon adaptation to the host was evaluated using the codon adaptation index (CAI) and the normalized arithmetic mean of codon frequency, which was calculated according to formula 1. Briefly, the abundance of each codon in an organism was calculated and pondered by a factor corresponding to its frequency in the whole genome. This value was normalized by the minimum and maximum values of a theoretical gene encoding for the same protein. A theoretical gene with a maximum normalized arithmetic mean of codon frequency always uses the codon with the highest frequency for codifying amino acids, while a theoretical gene with a minimum normalized arithmetic mean always uses the codon with the lowest frequency. In formula 1, for a codon “i”, “F_i_” represents its codon frequency in the genome, and “f_i_” represents its absolute frequency in the analyzed gene. For an amino acid “a”, Z_a_ represents its absolute frequency in the encoded protein, while F_amin_ and F_amax_ represent the minimum and maximum genomic codon frequency for the codon corresponding to amino acid “a”. We considered the number of amino acids as 20, without taking in account the 3 codons that encode for a stop codon.

$$\mathrm{NormalizedMeanCodonFrequency}=\frac{\left(\sum_{i=1}^{i=61}\left(F_{i}*f_{i}\right)-\sum_{a=1}^{a=20}\left(Z_{a}*F_{a\min}\right)\right)}{\left(\sum_{a=1}^{a=61}\left(Z_{a}*F_{\text{amax}}\right)-\sum_{a=1}^{a=20}\left(Z_{a}*F_{a\min}\right)\right)}$$

**Formula 1**: Calculation of the normalized arithmetic mean of codon frequency.

The codon frequencies for *Salmo salar* and *Homo sapiens* were obtained from the Kazusa database (http://www.kazusa.or.jp/codon/). In both cases, the CAI was calculated with EMBOSS software [49], and the normalized arithmetic mean of codon frequency was calculated with a script in Python2.6 (Additional file 3, and Additional file 4). CAI and normalized arithmetic mean of codon frequency values for viral genes were calculated using the codon frequencies of the host organism. Statistical parameters were calculated using Sigma Plot 10.0. Correlation analyses were performed using the non-parametric Spearman rank order correlation test. Under this test p-values greater than 0.050 indicate there is no significant relationship between two given variables. Bonferroni correction was applied when multiple correlations were performed [50]. In order to compare CAI and the normalized arithmetic mean of codon frequency values of genes from viruses that infect different hosts, Z values were developed that normalize the CAI and normalized arithmetic mean of codon frequency values of viral genes with respect to the values of cellular genes. The Z values were calculated as (X_I,O_ – mean_O_)/σ_O_, where X_I,O_ represents the CAI or normalized arithmetic mean of codon frequency values of a gene “I” from a virus that infects an organism “O”, and mean_O_ and σ_O_ represent the mean and standard deviation of the CAI or normalized arithmetic mean of codon frequency values of all nuclear genes from the organism “O”. Gene ontology classification was performed with Blast2GO software [51]. Briefly, classifications were made with standard parameters: e-value hit filter = 10^-6^, annotation cut-off = 55 and GO-weight = 5. To make the association between CAI and the GO code, the gi, CAI, GO codes and classification types (whether by biological process, molecular function or cellular component) for each gene were tabulated. We then determined the number of biological processes, molecular functions and cellular components of genes with CAI values of 0.01 units of the CAI value of the analyzed viral genes. The percentage of genes was calculated in function of the total GO terms identified for each biological process, molecular function or cellular component. Mortality levels of the ISA virus were taken from Mjaaland et al. [36]. Graphs with color coding were prepared using MeV multiexperiment viewer [52]. Phylogenetic relationship between ISAV genes were previously published [46], briefly this phylogenies were estimated using Bayesian Inference as implemented in BEAST 1.5.3, with a relaxed uncorrelated lognormal molecular clock. Bayesian posterior probabilities were determined by running 200 million generations, and the trees were sampled every 20,000 generations.

## Competing interests

The authors declare that they have no competing interests .

## Authors’ contributions

MT designed all *in silico* experiments. FV performed all computational analysis and calculations. ES contributed to data analysis and wrote the manuscript. All authors read and approved the final manuscript.

## Supplementary Material

Additional file 1 Table S1 This is a Microsoft Excel document containing supplementary tables about Z-CAI values from ISAV genes (Additional file 1: Table S1), CAI and Z-CAI values from viral pathogens of *Salmo salar* (Additional file 1: Table S2), Z-CAI values from Orthomyxovirus genes (Additional file 1: Table S3), values of the normalized mean of codon frequency in ISAV genes (Additional file 1: Table S4), Z values of NMCF (normalized mean of codon frequency) from Orthomyxovirus genes (Additional file 1: Table S5), Z values of NMCF (normalized mean of codon frequency) from ISAV genes (Additional file 1: Table S6) and Z values of NMCF (normalized mean of codon frequency) from viral pathogens of *Salmo salar* (Additional file 1: Table S7). Click here for file

Additional file 2 Figure S1 This is a Microsoft Word document containing supplementary figures about host and Orthomyxovirus genes classified according to their cellular process and CAI values (Additional file 2: Figure S1), correlations between codon adaptation of ISAV genes (Additional file 2: Figure S2), correlations between CAI and normalized mean codon frequency values of *Salmo salar* genes (Additional file 2: Figure S3) and comparison of normalized means of codon frequency (NMCF) values from segments of closely related ISAV (Additional file 2: Figure S4). Click here for file

Additional file 3 Scripts written in python language to calculate the normalized mean codon frequency of a coding region in FASTA format. Click here for file

Additional file 4 This file explains how to use the script to calculate the normalized mean of codon frequency. Click here for file
