# Supplementary material for: Genomic adaptation of the ISA virus to Salmo salar codon usage
Source: Virol J. 2013 Jul 5;10:223. doi: 10.1186/1743-422X-10-223 (PMC3706250; doi:10.1186/1743-422X-10-223)
Supplement: Additional file 2 Figure S1 — This is a Microsoft Word document containing supplementary figures about host and Orthomyxovirus genes classified according to their cellular process and CAI values (Additional file 2: Figure S1), correlations between codon adaptation of ISAV genes (Additional file 2: Figure S2), correlations between CAI and normalized mean codon frequency values of Salmo salar genes (Additional file 2: Figure S3) and comparison of normalized means of codon frequency (NMCF) values from segments of closely related ISAV (Additional file 2: Figure S4). [file 1743-422X-10-223-S2.doc]

**Supplementary Figures**

Supplementary Figure 1:

**
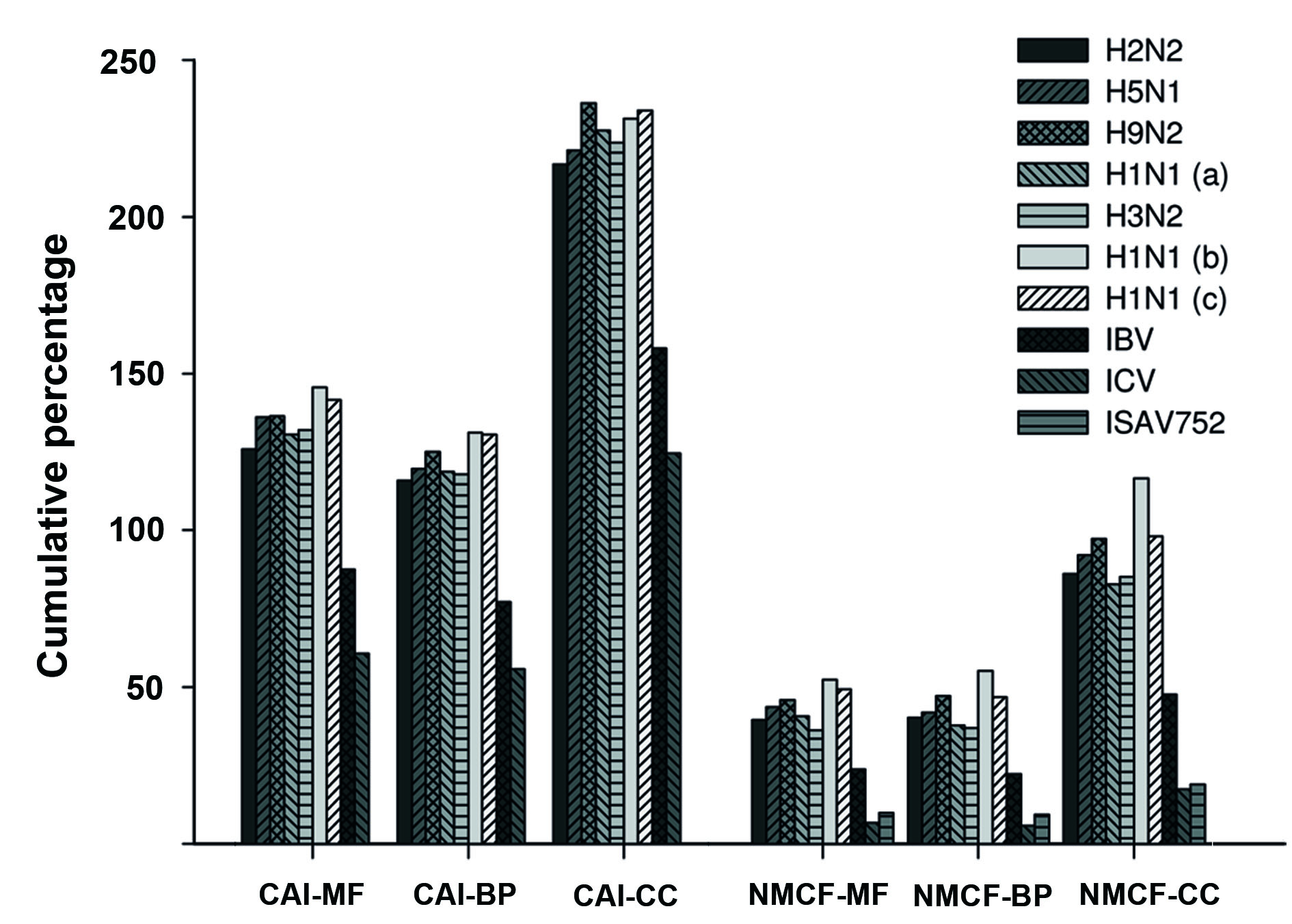
**

**Supplementary Figure 1: Mimicking cellular processes:**. The *Salmo salar* and human genes were classified according to GO (see Methods). Using this classification, we determined the number of molecular functions (MF), biological processes (BP) and cellular components (CC) that contain genes with CAI values or normalized mean codon frequency (NMCF) values similar those in ISAV or influenza genes. For each virus the cumulative percentage is the sum of percentages of cellular processes determined for the viral genes. (a) H1N1 2009, (b) H1N1 1918 and (c) H1N1 1934. The Chilean isolate ISAV752 was selected for the analyses.

Supplementary Figure 2


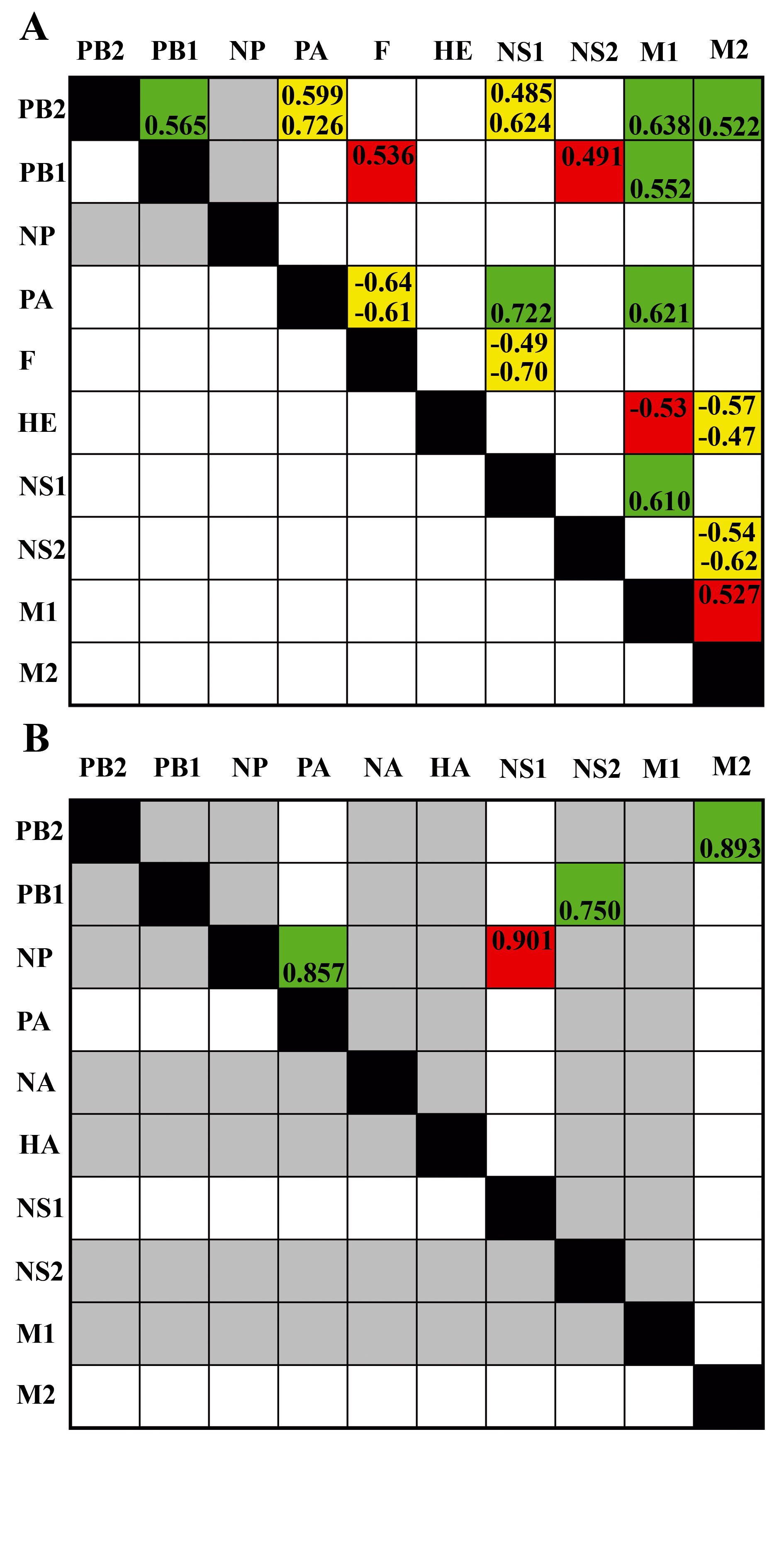


**Supplementary Figure 2: Correlation between codon adaptation of ISAV genes**: The figure shows the correlation between codon adaptation of genes from ISA virus (n = 18). The statistical correlations (P < 0.05) among genes using the CAI or normalized mean of codon frequency are shown in red and green squares, respectively. Yellow squares indicate a statistical correlation using both indices. The correlation coefficients (ρ) using CAI (upper number) or the normalized mean of codon frequency (lower number) are also shown.


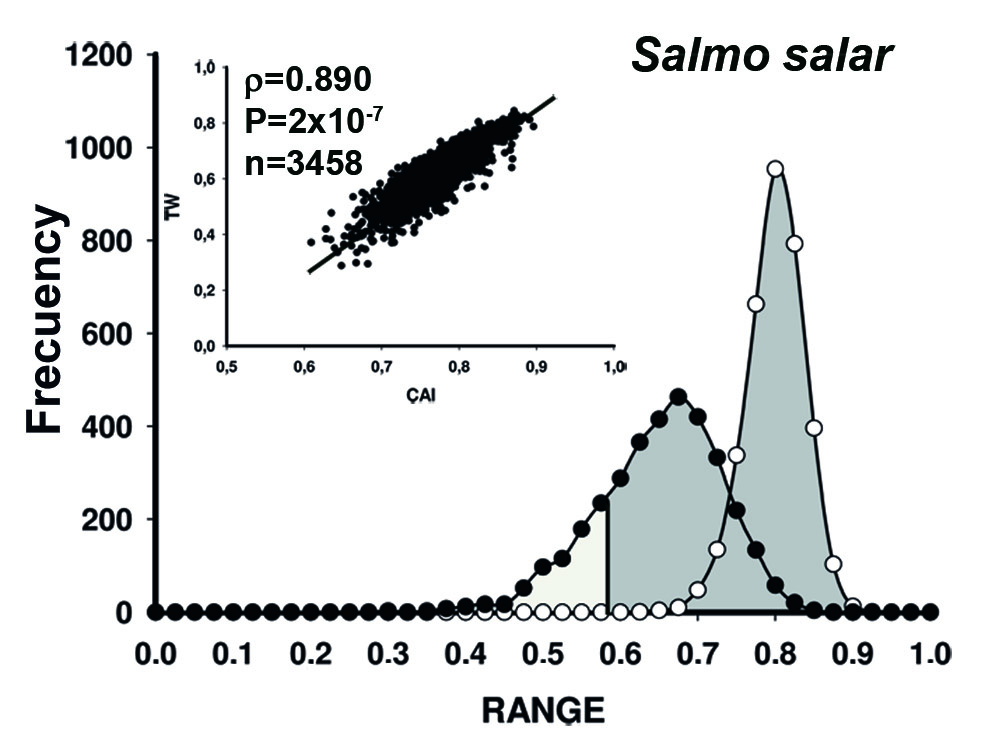


**Supplementary figure 3**: **Correlation between CAI and the normalized mean of codon frequency values in genes from *Salmo salar***

The histogram of the frequency of CAI and the normalized mean of codon frequency (NMCF) values of the *Salmo salar* genes. The inner graph shows the correlation between CAI and NMCF values in the *Salmo salar* genome. The white area shows the proportion of *Salmo salar* genes with NMCF values similar to those in ISAV.


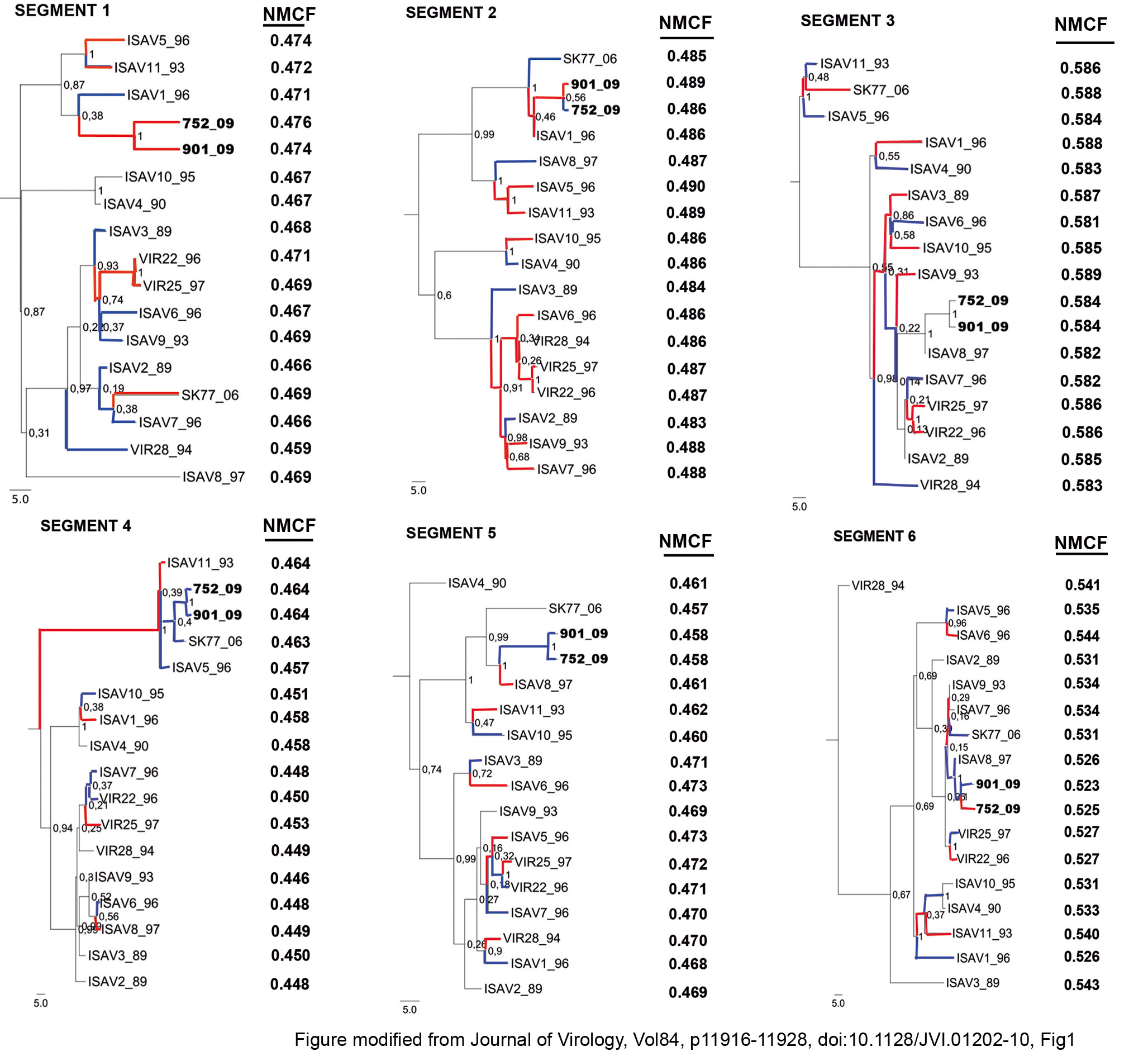


**Supplementary figure 4:** **Comparison of normalized mean of codon frequency (NMCF) values from segments of closely related ISAV.** The alignment was taken from a previous work of our group [44]. Red lines show the divergence that increases the NMCF value and the blue lines show the divergence that decreases NMCF values.
