# Supplementary material for: Genomic adaptation of the ISA virus to Salmo salar codon usage
Source: Virol J. 2013 Jul 5;10:223. doi: 10.1186/1743-422X-10-223 (PMC3706250; doi:10.1186/1743-422X-10-223)
Supplement: Additional file 4 — This file explains how to use the script to calculate the normalized mean of codon frequency. [file 1743-422X-10-223-S4.doc]

The NMCF.py script allows for calculating the normalized mean of codon use frequency. The script is written in Python programming language and rquires three files:

1. A file in FASTA format with codifying sequences of the genes to calculate the NMCF value.

2. A file with the frequency of the host codon use “**tabla_uso2.frec**”, written in the following format:

CGT 18.6 2495 R

CGC 10 1347 R

CGA 4 539 R

CGG 6.6 889 R

AGA 10.1 1360 R

AGG 13.4 1801 R

TCT 13.9 1863 S

TCC 21.2 2848 S

TCA 10.1 1364 S

TCG 5.3 714 S

AGT 11.2 1506 S

AGC 21 2824 S

TTA 3.4 462 L

TTG 10.2 1366 L

CTT 7.9 1058 L

CTC 20.8 2794 L

CTA 7.2 965 L

CTG 46 6183 L

CCT 13.9 1868 P

CCC 20.4 2739 P

CCA 13.3 1788 P

CCG 5.9 797 P

GCT 17.5 2352 A

GCC 26.9 3614 A

GCA 13.4 1804 A

GCG 7.2 968 A

ATC 29.2 3922 T

ACC 24 3223 T

ACA 14.7 1975 T

ACG 6.9 929 T

GGT 14 1886 G

GGC 22.3 2998 G

GGA 20.2 2721 G

GGG 15.9 2134 G

GTT 9.8 1314 V

GTC 18.6 2495 V

GTA 6.2 832 V

GTG 29.1 3906 V

ATT 11.3 1525 I

ATC 29.2 3922 I

ATA 6.4 856 I

CAT 8.6 1160 H

CAC 18 2417 H

TAT 8.1 1091 Y

TAC 21.9 2920 Y

AAA 17.1 2300 K

AAG 39.7 5335 K

AAT 11.4 1535 N

AAC 28.8 3878 N

TTT 12.5 1687 F

TTC 26 3498 F

GAT 16.8 2253 D

GAC 34.5 4640 D

TGT 11 1485 C

TGC 12.6 1689 C

CAA 8.3 8.3 Q

CAG 36 4835 Q

GAA 13.9 1866 E

GAG 10.1 1360 E

TGG 11.5 1550 W

ATG 27.3 3666 M

Where the first column is the codon, the second is the frequency of codon use, the third column is the codon count and the fourth is the amino acid that the codon encodes.

3. A file with the minimum and maximum values of codon use “**tabla_maxmin2.frec**” for each amino acid. The file should be written in the following format:

4 18.6 R

5.3 21.2 S

3.4 46 L

5.9 20.4 P

7.2 26.9 A

6.9 29.2 T

14 22.3 G

6.2 29.1 V

6.4 29.2 I

8.6 18 H

8.1 21.9 Y

17.1 39.7 K

11.4 28.8 N

12.5 26 F

16.8 34.5 D

11 12.6 C

8.3 36 Q

10.1 13.9 E

11.5 11.5 W

27.3 27.3 M

where the first column is the minimum frequency, the second column is the maximum frequency and the third column is the amino acid.

The tables used as examples are the codon use frequencies in Salmo salar. The frequency of use for other organisms can be obtained from [www.kazusa.com](http://www.kazusa.com/)
